# Fertility Preservation for Adolescent and Young Adult Transmen: A Case Series and Insights on Oocyte Cryopreservation

**DOI:** 10.3389/fendo.2022.873508

**Published:** 2022-05-24

**Authors:** Francesca Barrett, Jacquelyn Shaw, Jennifer K. Blakemore, Mary Elizabeth Fino

**Affiliations:** ^1^ Department of Obstetrics and Gynecology, New York University Grossman School of Medicine, New York, NY, United States; ^2^ Department of Reproductive Endocrinology and Infertility, New York University Langone Fertility Center, New York, NY, United States

**Keywords:** transgender, adolescent reproductive health, oocyte cryopreservation, adolescent and young adult (AYA), fertility, fertility preservation

## Abstract

**Background:**

The opportunity for fertility preservation in adolescent and young adult (AYA) transmen is growing. Many AYA transmen desire future biologic children and are interested in ways to preserve fertility through oocyte cryopreservation prior to full gender affirmation, yet utilization of oocyte cryopreservation remains low. Additionally, standard practice guidelines currently do not exist for the provision of oocyte cryopreservation to AYA transmen. Our objective was to review our experience with oocyte cryopreservation in adolescent and young adult transmen in order to synthesize lessons regarding referral patterns, utilization, and oocyte cryopreservation outcomes as well as best practices to establish treatment guidance.

**Methods:**

This is a case series of all AYA transmen (aged 10 to 25 years) who contacted, consulted or underwent oocyte cryopreservation at a single high volume New York City based academic fertility center between 2009 and 2021.

**Results:**

Forty-four adolescent and young adult transmen made contact to the fertility center over the study period. Eighty percent (35/44) had a consultation with a Reproductive and Endocrinology specialist, with a median age of 16 years (range 10 to 24 years) at consultation. The majority were testosterone-naive (71%, 25/35), and had not pursued gender affirming surgery (86%, 30/35). Expedited initiation of testosterone remained the most commonly cited goal (86%, 30/35). Fifty-seven percent (20/35) pursued oocyte cryopreservation. Ninety-five percent (19/20) underwent successful transvaginal oocyte aspiration, with a median of 22 oocytes retrieved and 15 mature oocytes cryopreserved. There were no significant adverse events. At time of review, no patient has returned to utilize their cryopreserved oocytes.

**Conclusions:**

Oocyte cryopreservation is a safe fertility preservation option in AYA transmen and is an important aspect of providing comprehensive transgender care. Insights from referral patterns, utilization, and oocyte cryopreservation outcomes from a single center’s experience with adolescent and young adult transmen can be integrated to identify lessons learned with the goal of providing transparency surrounding the oocyte cryopreservation process, improving the education and comfort of patients and providers with fertility preservation, and easing the decision to pursue an oocyte cryopreservation cycle in parallel to gender-affirmatory care.

## Introduction

The prevalence of transgender and gender-diverse adults has doubled over the past decade, with prevalence of AYA transgender youth estimated to be between 0.6% to 3.2% of the U.S population (1–4). Adolescents identify as transgender more often than adults, and gender affirming treatments are occurring at younger ages, often as AYA (ages 10 to 25 years) (1–4). National and international organizations recommend fertility preservation counseling regarding oocyte cryopreservation, embryo banking, and ovarian tissue cryopreservation for transgender male individuals prior to initiating gender-affirming treatments (5–7). Many AYA transmen desire future biologic children and are interested in ways to preserve their fertility (8–14). Fertility preservation has primarily been through oocyte cryopreservation, which is a safe and feasible option that does not require a partner or use of donor sperm (9–14). Despite increasing prevalence, societal support, and interest in fertility preservation, utilization of oocyte cryopreservation remains low in AYA transmen, with several small studies reporting rates between 0% to 7.8% (15–19).

Gaps in utilization of fertility preservation for AYA transmen are multifactorial (20, 21). Some AYA patients prioritize their desire to initiate testosterone as soon as possible and thereby forego fertility preservation and its associated counseling (15, 16, 22). Others defer consultation due to fear surrounding the invasiveness of fertility preservation procedures or potential gender dysphoric triggers (9, 15, 16, 21, 23). There are financial barriers due to the high cost of services often uncovered by insurance as well as systems barriers in the form of primary care providers who are inexperienced with AYA transmen’s fertility needs or have difficulty counseling patients based on current fertility preservation outcomes research (9, 15, 16, 23–25). Others consider parenthood with biologic children a low priority during their adolescence (21).

Furthermore, there are no standard practice guidelines for the provision of oocyte cryopreservation to AYA transmen (6). Lack of standardized fertility preservation practices for AYA transmen reinforces these gaps by reducing transparency surrounding fertility preservation and precluding patient and provider education (22). As a result of low utilization, research on the experiences and outcomes of oocyte cryopreservation within AYA transmale population is limited. Current data are primarily from case reports and small case series reporting general findings surrounding gonadotropin requirements, hormonal levels during stimulation, and mature oocyte retrieval yields (10, 26–31).

Therefore, we performed a case series of AYA transmen interested in fertility preservation to evaluate referral patterns, utilization, and oocyte cryopreservation outcomes to identify important care points. Our objective was to synthesize experiences across patients who contacted, consulted, and underwent oocyte cryopreservation to define lessons learned and best practices for fertility preservation amongst AYA transmen.

## Materials and Methods

### Design

A case series was performed between October 2009 and June 2021 of all AYA transmen who made contact with New York University Langone Fertility Center (NYULFC), a high volume New York City based academic fertility center capable of oocyte cryopreservation and embryo banking for fertility preservation. This study was performed with New York University Grossman School of Medicine Institutional Review Board approval (i13-00389).

### Subjects

All AYA subjects, defined as individuals between the ages of 10 and 25 years old, who contacted NYULFC during the study period were reviewed. Patients were included if documentation confirmed they identified as transmale or gender non-conforming individuals assigned female at birth. Patients were excluded if 1) they identified as transfemale or gender non-conforming individuals assigned male at birth, or 2) they were younger than 10 or older than 25 years at time of initial consultation.

### Variables and Data Collection

Electronic medical records were reviewed to extract all demographics and outcome variables. All records were reviewed for referral source (provider and institution) and age at contact. Records of AYA transmen who proceeded with consultation with a Reproductive Endocrinology and Infertility (REI) physician were reviewed for age at consultation, age at initiation of gender affirmation through reported through pronoun changes, breast binders, or other social changes, pre-consultation gender affirming treatments including menstrual suppression, testosterone, gender affirming surgery, and hormonal implants, post-consultation gender affirming treatments, and goals at time of consultation. Records of AYA transmen who proceeded with fertility preservation were reviewed for assisted reproductive technology outcomes, including baseline hormonal labs, ultrasound modality for monitoring, stimulation dosing, trigger dosing, days of stimulation, number of oocytes retrieved, number of mature and immature oocytes frozen, total number of cycles, oocyte disposition, adverse events, and documentation of practices to minimize dysphoria. Episodes of gender dysphoria were assessed by reviewing documentation of pre/post procedure progress notes described by REIs, social workers, and/or primary care providers. Records of AYA patients who did not proceed with fertility preservation were reviewed for post-contact/consultation gender affirming treatments and reported reasons for deferral that were documented within the medical chart to REIs, social workers, and/or primary care providers following consultation. The primary outcome was number of mature oocytes cryopreserved. Secondary outcomes included number of immature oocytes cryopreserved, utilization of consultation and fertility preservation, rates of reported cycle-related gender dysphoria, initiation of gender affirming treatments following contact, consultation, and fertility preservation, rates of low trigger response and cancelled cycles, and rates of adverse outcomes.

### Ovarian Stimulation and Oocyte Cryopreservation

Two protocols were utilized for controlled ovarian hyperstimulation: a gonadotropin-releasing hormone antagonist protocol and a low-dose down-regulation protocol with leuprolide acetate. Protocols were prescribed to each subject per provider discretion. Subjects received gonadotropins (recombinant follicle-stimulating hormone, human menopausal gonadotropins, or both) for all protocols, with follicular growth monitored by serial serum estradiol levels and ultrasound monitoring. For antagonist protocols, the gonadotropin-releasing hormone antagonist was initiated when 1) a lead follicle was identified as 13 mm^2^; 2) the estradiol was >1,000 pg/mL; or 3) at the discretion of the primary provider. Either human chorionic gonadotropin (hCG) alone or with leuprolide acetate, as appropriate, was used for the trigger of final oocyte maturation. Oocyte aspiration was scheduled for 35 hours after trigger administration as is routine/standard at our center. Retrievals were performed *via* ultrasound-guided transvaginal aspiration. Oocytes were cryopreserved *via* slow frozen methodology or *via* vitrification, as was standard of care in the embryology laboratory at the time of cryopreservation using previously described techniques (32).

### Analysis

Continuous variables were assessed for normality using Kolmogorov-Smirnoff test and determined to be non-parametric; thus Mann-Whitney tests were used to compare continuous variables. Categorical variables were analyzed using the Chi-squared tests or Fisher’s exact, where appropriate, to assess for group differences in survey measures by demographic and professional characteristics. An alpha error of 0.05 was considered statistically significant. Descriptive results are reported as percent, counts, median, and range. Key themes and lessons were identified for areas of excellences and gaps.

## Results

### Referrals and Utilization

**Figure 1:**
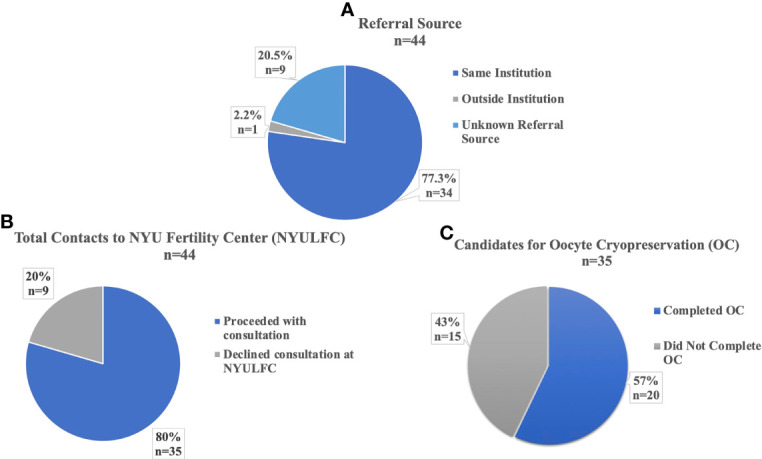
Referrals and Utilization of AYA transmen at NYU Langone Fertility Center. **(A)** Institutional Source for Referral to NYU Fertility Center. **(B)** Percent of Total Contacts to NYU Fertility Center who Proceeded with Consultation. **(C)** Percent of Consultations who were Candidates for Oocyte Cryopreservation.

A total of 44 AYA transmen contacted the NYULFC between October 2009 and June 2021 with a median age of 17 years (range 10-24 years) at time of contact. Most referrals came from providers within the same institution (77%, 34/44), with two providers (a pediatric adolescent physician and a psychologist) from the Gender and Sexuality (G&S) service at the NYU Hassenfeld Children’s Hospital referring the majority of patients (**Figure 1A**). Other referring providers included in-institution psychiatrists/psychologists, an urologist, an endocrinologist, a pediatrician, and a plastic surgeon and a gynecologist that both perform gender affirming surgery. Only one patient was referred from an outside institution (**Figure 1A**).

Eighty percent (35/44) of patients who contacted the fertility center proceeded with consultation with a REI physician (**Figure 1B**). Nine percent (3/35) of consultations occurred before 2017, with 69% (24/35) of consultations between 2019-2021. Ninety-four percent (33/35) of patients who consulted with a REI were eligible for oocyte cryopreservation (OC). The two ineligible patients were aged 10 and 13 years and were Tanner stage II; both were encouraged to return at later development stages for OC or consider tissue cryopreservation at another institution, as ovarian tissue cryopreservation was not offered at our center in the study period. Fifty-seven percent (20/35) of consulting patients pursued OC (**Figure 1C**). No patients pursued embryo cryopreservation. Patients proceeded quickly with OC following consultation, with 73% (16/22) of cycles starting within two months of consultation, with median one month (range 1-17 months) between consultation and cycle start date. Median age at consultation was 16 years (range 10-24 years) and median age at cycle start was 16 years (range 12-25 years).

Records were available for all patients who deferred consultation with a REI physician, though documentation of reason for deferral was limited (**Table 1**). Most patients that deferred consultation were on testosterone before or initiated shortly after initial contact to the fertility center with all reporting awareness of the possible fertility impact. Five patients expressed certainty that they were not interested in genetic children. One patient underwent oocyte cryopreservation at a different fertility center and subsequently started testosterone. Another patient was concerned fertility preservation would be too distressing and opted to begin pubertal suppression with leuprolide acetate.

**Table 1:** Characteristics of AYA transmen who did not seek consultation.

Age Range at Contact (years)	Initiation of Testosterone prior to consultation	Reason for declining consultation	Clinical Next Steps
16-18	No	Not interested in biologic offspring	Began Testosterone
			Oocyte cryopreservation at alternative institution
			Began testosterone
16-18	No	Oocyte cryopreservation at different institution	Underwent gender affirming top surgery
			Began leuprolide acetate
13-15	No	Concern for dysphoria with fertility preservation	Underwent gender affirming top surgery
19 and older	Yes	Not interested in biologic offspring	Underwent gender affirming bottom surgery and phalloplasty
19 and older	Yes	Not interested in biologic offspring	Continued testosterone
19 and older	No	Not interested in biologic offspring and desire to initiate testosterone	Began testosterone
19 and older	Yes	Not indicated	Continued testosterone
16-18	No	Desire to initiate testosterone	Began testosterone
13-15	Yes	Not interested in biologic offspring	Continued testosterone

### Consultation Characteristics and Goals

General characteristics for those who sought consultation can be seen in **Table 2**. At time of consultation, patients were at varying stages of their gender affirmation. Most patients were earlier in their gender affirmation, as the majority were testosterone-naive (71%, 25/35), and had not pursued gender affirming surgery (86%, 30/35). Patients reported differing primary goals for their consultation including fertility preservation prior to testosterone or gender affirming surgery (13/35), expedited initiation of testosterone (9/35), initiation of testosterone alongside other expedited gender affirming treatments (8/35), family building (2/35), menstrual suppression (1/35), or not documented (2/35). Only seven patients expressed certainty of their desire for a genetic child, though many were concerned that they might miss the opportunity to preserve fertility prior to starting definitive treatments. Several patients’ goals for affirmation outweighed their goals for fertility preservation. Of the patients who did not seek oocyte cryopreservation following consultation, four patients underwent gender affirming top surgery, one patient underwent gender affirming bottom surgery, and four patients began testosterone though reported desire to complete fertility preservation in the future. In comparison, several patients who prioritized fertility preservation pursued gender affirming treatments after completing oocyte cryopreservation, including twelve testosterone naïve patients initiating testosterone and nine patients undergoing their first gender affirming surgery.

**Table 2:** Characteristics of AYA transmen who underwent fertility preservation consultation with a Reproductive Endocrinology and Infertility physician.

	Declined Fertility Preservation	Underwent Oocyte Cryopreservation	P value
	n = 15	n = 20	
**General Characteristics of Referrals**			
Age at consultation [years, median (range)]	18 (10-24)	16 (12-23)	0.16
Age at gender identity affirmation [years, median (range)]	12 (9-17)	14 (11-21)	0.56
Time between Gender Affirmation and Consult [years, median (range)]	2 (1-13)	2 (1-4)	0.56
**Pre-Consultation Gender Affirming Treatments**			
Menstrual suppression with oral contraception	27% (4/15)	15% (3/20)	0.31
Pubertal blocker with leuprolide acetate	20% (3/15)	10% (2/20)	0.63
Testosterone	33% (3/15)	10% (2/20)	0.63
Gender Affirmatory Top Surgery	27% (4/15)	5% (1/20)	0.14
**Post-Consultation Gender Affirming Treatments**			
Menstrual suppression with oral contraception	27% (4/15)	15% (3/20)	0.31
Menstrual suppression with leuprolide acetate	27% (4/15)	10% (2/20)	0.39
GNRH implant	0% (0/15)	5% (1/20)	1.00
Testosterone	47% (7/15)	70% (14/20)	0.69
Gender Affirmatory Top Surgery	53% (8/15)	45% (9/20)	0.28
Gender Affirmatory Bottom Surgery	7% (1/15)	5% (1/20)	1.00
**Fertility Goals**			
Unsure of desire of future biological children (%)	53% (8/15)	55% (11/20)	0.67
Certain of desire of future biological children (%)	13% (2/15)	25% (5/20)	
No documentation of desire of future biologic children (%)	33% (5/15)	20% (4/20)	

### Dysphoria Protection Protocols

Care was taken to minimize gender dysphoric events during consultation, ovarian stimulation, and oocyte retrieval, in accordance with World Professional Association for Transgender Health (WPATH) “Standards of Care for hormone-prescribing physicians for the Health of Transsexual, Transgender, and Gender Nonconforming People,” which outlines the specific responsibilities of hormone-prescribing physicians (5). At the initial consultation, alongside pertinent discussion of patient’s affirmation goals, health history, physical exam, and relevant laboratory tests, patients were asked preferred language for first name, pronouns, as well as possible triggering gendered terms, including but not limited to menstruation, female organs, and oocytes/eggs. No patient underwent pelvic examination at initial consultation per patient preference.

During ovarian stimulation, patient’s medical records had name alerts to ensure patients were called by appropriate preferred names and pronouns. Ovarian stimulation ultrasound monitoring was completed transabdominally for 82% (18/22) of cycles. Of the remaining four cycles, transvaginal ultrasound was utilized to assess ovarian pathology at initial scan (2/22), to visualize ovaries that were unable to be seen abdominally (1/22), or to accommodate patient preference for vaginal monitoring (1/22). No patients requiring transvaginal monitoring had been on testosterone and no dysphoric events were recorded related to transvaginal monitoring. There were no documented events of patients experiencing gender dysphoria specifically related to ovarian stimulation.

For oocyte retrieval procedures, patients were allowed to maintain chest binder and wear undergarments in the operating room until fully sedated if so desired. Patients were positioned into dorsal lithotomy only after deep sedation was achieved. All oocyte aspirations were completed transvaginally. Patients were counseled preoperatively on possibility of hymenectomy at time of retrieval to accommodate the transvaginal ultrasound probe, but no hymenectomies were required. Undergarments were replaced prior to patient’s movement to the recovery area. Special accommodations were encouraged during the recovery period, such as private recovery area or inclusion of parents if desired during recovery. There were no documented events of patients experiencing gender dysphoria during the oocyte retrieval or in the postoperative period.

### Fertility Preservation Outcomes

Individual outcomes for oocyte cryopreservation can be seen in **Table 3A** and cumulative summary characteristics in **Table 3B**. There were a total of 20 patients who attempted oocyte cryopreservation, 19 patients who underwent oocyte retrieval, and two patients who completed two cycles of oocyte cryopreservation, for a sum total of 21 completed oocyte cryopreservation cycles and one cancelled cycle. Median age at cycle start was 16 years (range 12-25 years). Baseline anti-mullerian hormone (AMH) level was median 3.26 ng/ml (range 0.44-12.87 ng/ml). 95% (21/22) of cycles had unmedicated cycle day two starts. Median cycle day two follicle stimulating hormone (FSH) was 5.65 mIU/mL (range 1.7-9.5 mIU/mL) and cycle day two estradiol (E2) was 43 pg/mL (range 19-163 pg/mL). There was one random start cycle to expedite oocyte cryopreservation and no luteal start cycles. Ninety-five percent (21/22) of cycles were gonadotropin-releasing hormone antagonist protocols. Notably, one patient with a baseline luteinizing hormone (LH) level of 0.4 mIU/L, had serial LH level monitoring but never required administration of GnRH antagonist prior to retrieval. The remaining cycle utilized a low-dose down-regulation protocol with leuprolide acetate prior to administration of gonadotropins. Notably, no cycles included letrozole in the protocol. Median total gonadotropin dose was 2375 IU (range 825-8075 IU) over median 10 days of stimulation (range 8-21 days), with median initial dosages 150 IU FSH (range 50-300 IU) and 75 IU human menopausal gonadotrophin (HMG) (range 0-150 IU).

**Table 3A:** Oocyte Cryopreservation Outcomes for Individual AYA Transmale Cycles.

Age Range at gender affirmation (years)	Age Range at cycle start (years)	Pre-consultation gender affirming therapy	AMH (ng/ml)	Protocol	Total gonadotrophin dose (IU)	Duration of stimulation (days)	E2 max (pg/mL)	Trigger	Total oocytes retrieved	MII oocytes frozen	MI oocytes frozen	GV oocytes frozen
12 and under	12 and under		1.58	Antagonist	4050	10	4212	1000U hCG + double GnRH-a, 5000U hCG boost	13	3	8	1
				Antagonist	5400	13	5175	10,000U hCG + double GnRH-a	9	6	1	2
13-15	13-15	Leuprolide acetate	1.53	Antagonist	3700	11	4288	1000U hCG + double GnRH-a	26	5	0	20
13-15	13-15		3.10	Antagonist	1750	9	3049	1000U hCG + single GnRH-a	15	9	3	2
13-15	13-15		3.26	Antagonist	2350	9	3822	1000U hCG + double GnRH-a	22	16	1	4
ND	13-15		0.73	Antagonist	1850	9	1059	1000U hCG + double GnRH-a	5	5	0	0
				Antagonist	2825	9	3084	1000U hCG + double GnRH-a	15	8	6	1
13-15	13-15	Oral contraception	2.70	Antagonist	2100	9	4023	1000U hCG + double GnRH-a	25	20	0	5
13-15	13-15		4.64	Antagonist	3250	13	2045	1000U hCG + single GnRH-a	19	14	2	3
ND	13-15		3.27	Antagonist	2775	10	2344	5000U hCG + double GnRH-a	22	15	3	4
13-15	16-18		9.84	Antagonist	825	8	5040	1000U hCG + double GnRH-a	32	18	3	10
13-15	16-18		3.00	Antagonist	1725	8	2848	1000U hCG + double GnRH-a	26	19	0	6
13-15	16-18	Oral contraception	3.60	Antagonist	2400	9	4393	1000U hCG + double GnRH-a	22	18	0	4
13-15	16-18		12.87	Antagonist	1925	10	6091**	1000U hCG + double GnRH-a	43	14	3	20
13-15	16-18	Oral contraception	0.44	Antagonist	4500	11	2673	1000U hCG + double GnRH-a	9	8	1	0
ND	16-18		5.93	Antagonist	2325	12	3151	10,000U hCG	21	17	0	0
ND	16-18		0.59	Antagonist	4050	10	3288	1000U hCG + double GnRH-a	33	21	8	3
ND	16-18		3.27	Antagonist	1750	8	2864	1000U hCG + single GnRH-a	30	26	1	3
13-15	16-18		5.77	Antagonist	8075	21	943ˆ	–	–	–	–	–
16-18	19 and older		6.18	Antagonist	2550	10	4007**	1000U hCG + double GnRH-a	26	23	2	1
ND	19 and older	Testosterone + Leuprolide acetate	ND	Low-dose leuprolide acetate downregulation	1900	11	6969**	10,000U hCG	59	35	9	0
19 and older	19 and older	Testosterone	7.35	Antagonist	2175	11	2792**	1000U hCG + double GnRH-a	20	18	1	1

ND, not documented.

AMH, Anti-mullerian hormone.

hCG, human chorionic gonadotropin.

E2, Estradiol level.

Total oocytes, Total oocytes (MII, MI, GV).

MII, Metaphase II oocytes.

MI, Metaphase I oocytes.

GV, Germinal Vesicle oocytes.

GnRH-a, gonadotrophin releasing hormone agonist,

Antagonist, gonadotropin-releasing hormone antagonist protocol.

Low-dose leuprolide acetate downregulation = provera for 10 days, leuprolide acetate 10U starting day 6 of provera reducing to 5U on cycle day 2 with initiating of gonadotrophins.

**Denotes experienced ovarian hyperstimulation syndrome (OHSS).

ˆDenotes canceled cycle.

**Table 3B:** Oocyte Cryopreservation Outcomes for All AYA Transmale Cycles.

Baseline Hormonal Laboratory Values	Median (Range)
AMH (ng/ml)	3.26 (0.44-12.87)
FSH (mIU/mL), cycle day 2	5.65 (1.7-9.5)
E2 (pg/mL), cycle day 2	43 (19-163)
	
**Stimulation Details**	**Median (Range) or Percent (Count)**
Initial FSH Gonadotropin Dose (IU)	150 (50-300)
Initial HMG Gonadotropin Dose (IU)	75 (0-150)
Total Gonadotropin Dose (IU)	2375 (825-8075)
Duration of Stimulation (days)	10 (8-21)
Maximum E2 Level (pg/mL)	3220 (943-6969)
Number of Cancelled Cycles	5% (1/22)
Reason for Cancelled Cycle	Low Response
Cycle Protocol	
Antagonist	95% (21/22)
Low-dose leuprolide acetate downregulation	5% (1/22)
Trigger Details	
10,000 U hCG only	9% (2/22)
1000 U hCG + single GnRH-a	9% (2/22)
1000 U hCG + double GnRH-a	59% (13/22)
5000 U hCG + double GnRH-a	14% (3/22)
10,000 U hCG + double GnRH-a	4.5% (1/22)
Post Trigger LH level (mIU/mL)	82.9 (0.9-187.2)
LH < 20mIUm/mL	15.8% (3/19)
LH 20mIUm/mL - 40mIUm/mL	10.5% (2/19)
LH > 40mIUm/mL	73.7% (14/19)
Post Trigger hCG, level (mIU/mL)	55 (2.66-215)
hCG < 40mIU/mL	47% (9/19)
hCG ≥ 40mIU/mL	53% (10/19)
	
**Retrieval Outcomes**	**Median (Range) or Percent (Count)**
Total oocytes retrieved, number	22 (5-59)
<10 oocytes retrieved	14% (3/21)
10-19 oocytes retrieved	19% (4/21)
≥ 20 oocytes retrieved	67% (14/21)
Maturity Rate	73% (19%-100%)
Number of MII oocyte cryopreserved	15 (3-35)
<10 MII oocytes	33% (7/21)
10-19 MII oocytes	43% (9/21)
≥ 20 MII oocytes	24% (5/21)
Number of MI oocyte cryopreserved	2 (1-9)
Any MI cryopreserved	71% (15/21)
Number of GV oocyte cryopreserved	3 (1-20)
Any GV cryopreserved	81% (17/21)

AMH, Anti-mullerian hormone.

FSH, Follicle stimulating hormone.

hCG, Human chorionic gonadotropin.

E2, Estradiol level.

HMG, Human menopausal gonadotrophin.

Antagonist, Gonadotropin-releasing hormone antagonist protocol.

Low-dose leuprolide acetate downregulation, Provera for 10 days, leuprolide acetate 10U starting day 6 of provera reducing to 5U on cycle day 2 with initiating of gonadotrophins.

GnRH-a, Gonadotrophin releasing hormone agonist.

LH, Luteinizing hormone.

Total oocytes, Total oocytes (MII, MI, GV).

MII, Metaphase II oocytes.

MI, Metaphase I oocytes.

GV, Germinal Vesicle oocytes.

Eighty-six percent of cycles (19/22 cycles) were triggered with a combination of recombinant human chorionic gonadotropin (hCG, 1000U, 5000U, or 10,000U) and leuprolide acetate (single 40U dose or two 40U doses 12 hours apart). Five patients had a low response to the combination trigger (LH < 40mIUm/mL) with one patient (LH level 0.9 mIU/mL and HCG level 21mIU/mL), requiring an additional hCG boost of 5000U while maintaining standard 35 hour post initial trigger retrieval time.

Two patients were on testosterone prior to initiation of cycle and resumed testosterone following OC (**Table 3A**). Both were weaned off of testosterone prior to cycle start date, one patient for three months and the other for two months. One patient with polycystic ovarian syndrome was cancelled for low response with a maximum estradiol 943 pg/mL and two dominant follicles after 21 days of stimulation. This patient did not proceed with an additional oocyte cryopreservation cycle following cancellation, but proceeded to initiation of testosterone.

A median of 22 oocytes (range 5-59) were retrieved. 67% of cycles (14/21) had at least 20 oocytes retrieved and cryopreserved. Most patients cryopreserved at least 10 mature oocytes (67%, 14/21) with many patients additionally freezing immature oocytes. All retrievals resulted in frozen Metaphase II (MII) oocytes (median 15, range 3-35). Two patients (age range 12-14 years) completed second oocyte cryopreservation cycles due to first cycle low maturity rate (23% MII, 3/13) and poor response (5 MII retrieved). Nine additional oocytes (6/9 MII oocytes) were cryopreserved for the first individual and 15 additional oocytes (8/15 MII oocytes) were cryopreserved for the second individual in the second oocyte cryopreservation cycle.

### Complications

Four cycles were complicated by mild or moderate ovarian hyperstimulation syndrome (OHSS) (**Table 3A**). All patients were managed with outpatient supportive care. Two of the patients had the highest maximum E2 (6969 pg/mL, 6091 pg/mL) and highest oocytes retrieved (59 oocytes, 43 oocytes). Notably, both patients with a history of testosterone use prior to stimulation experienced OHSS. One of the patients was on the low-dose down-regulation protocol with leuprolide acetate who required a hCG only trigger.

### Subgroup Analysis

Patients who received oral contraception, leuprolide, and/or testosterone were sub-divided to evaluate if fertility outcomes differed in those who received pre-consult affirming therapy compared to those who did not. Patients who received oral contraception, leuprolide, and/or testosterone prior to oocyte cryopreservation were significantly older at time of stimulation than those who had not started these gender affirming treatments (p=0.02). There were no other significant differences between groups regarding days of stimulation, AMH, day two FSH or E2, maximum E2, oocyte maturity (MII, MI, GV, or total), number of oocytes retrieved, or total gonadotropin dose. Importantly, all patients with prior testosterone exposure were successful in ovarian stimulation and in cryopreserving mature oocytes. (**Supplemental Chart A**).

## Discussion

Oocyte cryopreservation is a safe and viable option for AYA transmen to preserve their fertility and is an important consideration for providing comprehensive care to the growing transgender youth population. This case series demonstrated several important facets to AYA transmale fertility preservation care including: 1) the importance of referral networks; 2) the desire for expedited gender affirming treatments with or without oocyte cryopreservation; and 3) the feasibility and safety of oocyte cryopreservation alongside dysphoria protecting protocols. Our results showed that in-network hospitals referrals were critical in capturing potentially interested AYA transmen who contacted, consulted, and underwent oocyte cryopreservation, many of whom were unsure of their future desire for biologic children and/or had already begun gender affirming treatments including gender affirming surgery, menstrual suppression with pubertal blockers or oral contraceptive pills, and/or testosterone. Expedited initiation of testosterone remained one of the most common goals regardless of age across patients, and often drove decision-making surrounding pursuit of fertility preservation. To our knowledge, this study is the largest published case series describing the experience of AYA transmen through their journey through oocyte cryopreservation and may provide a foundation for solidifying best practices for AYA transmen who are considering fertility preservation with oocyte cryopreservation.

Low prevalence of fertility preservation has been attributed to cost of treatment, concerns about discrimination, discontinuation or delay of gender-affirming hormonal therapy, or worsening gender dysphoria (15–17, 19, 33). Our cases shared similar barriers relating to discontinuation or delay of hormonal therapy, though many of our patients were still able to proceed with expedited gender affirming treatments following oocyte cryopreservation. Desire for family building and fertility have previously been demonstrated as valuable to many transgender individuals, though desire for biologic children may be lower in transgender youth with greater uncertainty as to whether this opinion will change in the future (12, 16, 19, 21, 33–35). Our cohort shared similar views for uncertainty surrounding biologic parenthood, with an overall lower percentage of those certain of their desire for genetic children (12, 16, 21, 29).

Previous studies have also found need for mental preparation and dysphoric triggers during cycling, though no overt episodes were cited during this current study. The process of oocyte cryopreservation can be a highly feminizing experience with the administration of hormones to increase endogenous estrogens, the possibility for feminizing effects of estrogens, the need to discontinue testosterone or other gender affirming hormonal treatments, and the resumption of menses before beginning the process (9, 23). Health care providers can alleviate distress discussing potential dysphoric events prior to cycle initiation, by using gender-neutral language and preferred pronouns and by incorporating supportive individuals such as friends, family members and partners into the process (23). While none of our patients utilized aromatase inhibitors and experienced minimal dysphoric events, letrozole, when taken during a cycle, can maintain low serum estradiol levels, minimize pubertal development, and prevent gender dysphoria symptoms (36, 37). Monitoring with transabdominal ultrasound can additionally temporize the feminizing character of oocyte stimulation in transmen, for which the majority of our patients opted (9, 16, 31). Using a random start approach for ovarian stimulation can enable patients to proceed with their cycles without needing a menstrual bleed, which may in turn reduce gender dysphoric triggers and expedite timing of cycle initiation (15, 38).

The effect of long-term general affirming testosterone on future reproductive capacity is largely unknown, with even less known about fertility in transgender individuals who have had puberty halted with GnRH agonists. In our testosterone cohort, we found no significant differences in fertility outcomes, though the number was very small. In comparison, current data on the impact of oocyte cryopreservation outcome in transmen remains mixed, with one study finding those previously exposed to testosterone having lower total oocytes retrieved and lower maximum E2 whereas another cohort study that transgender men had overnight increased number of oocytes retrieved but required elevated total gonadotrophin doses (26, 30). As this field grows, the understanding of the impact of testosterone will provide further counseling tools and options for fertility in older transgender individuals. There are no prior studies on the impact of pubertal suppression on fertility preservation outcomes.

**Figure 2:**
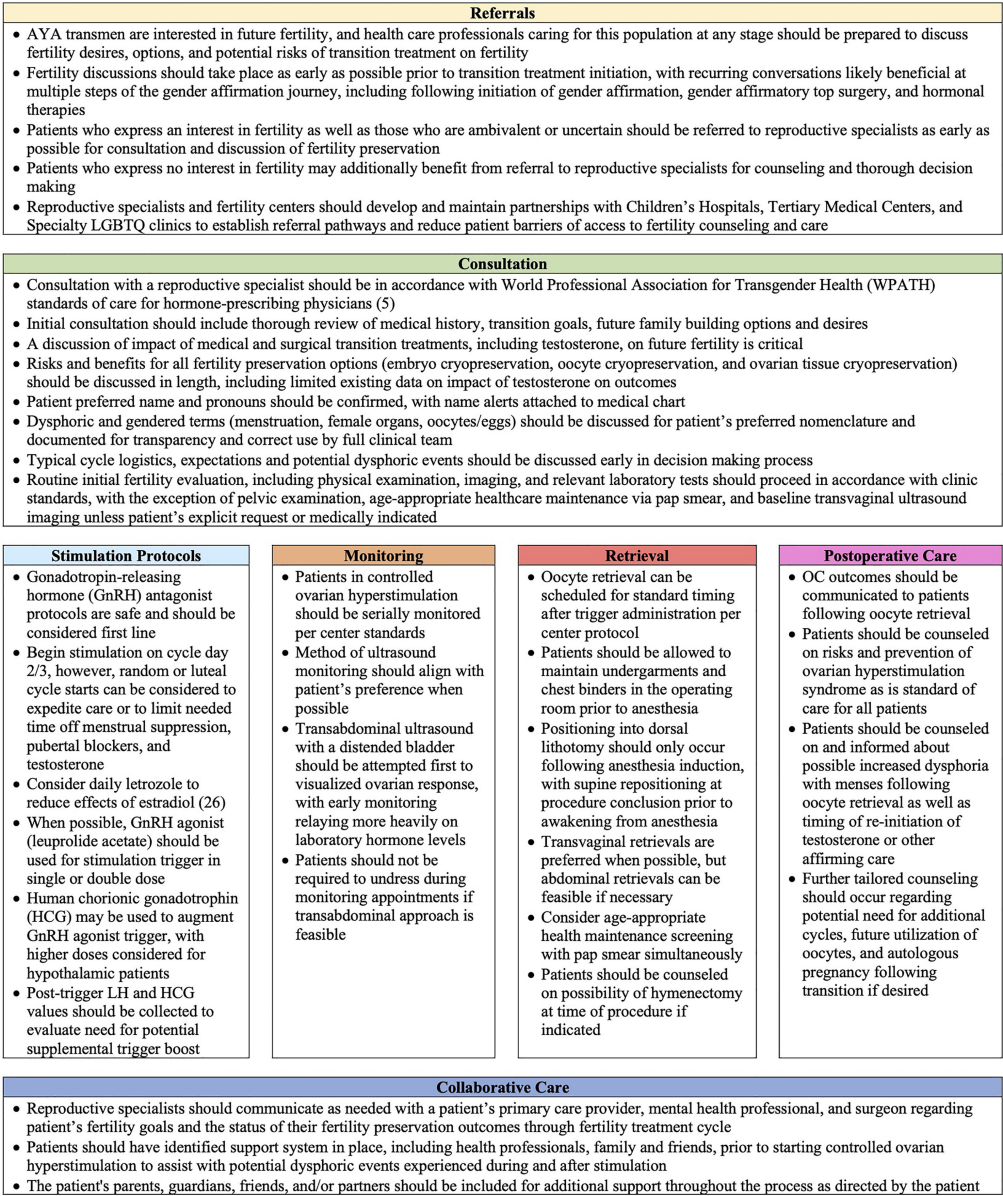
Lessons Learned from Adolescent and Young Adult Transmen Undergoing Oocyte Cryopreservation.

Utilizing the insights evaluated, we highlight key lessons learned for comprehensive care for AYA transmen who may consult or utilize oocyte cryopreservation, as seen in **Figure 2**. These recommendations are modeled from WPATH guidelines, but refined to focus on transparency of the process and specific actionable methodology for reproductive specialists to follow (5). AYA oncologic guidelines for fertility preservation were additionally reviewed for best practices that may be translatable for AYA transmen, including expedited care *via* random and luteal cycle starts and utilization of letrozole during ovarian stimulation to limit E2 to minimize pubertal development and prevent gender dysphoria symptoms (27–29, 37–40). We incorporate into our lessons medical ethics principles of nonmaleficence, beneficence, autonomy as well as the WPATH tenet for creating a safe and supportive environment to maximize the overall health, psychologic well-being, and self-fulfillment of transgender patients (5). Lessons learned are highlighted across seven main pillars for AYA transmen for best practices regarding fertility preservation and oocyte cryopreservation: 1) Referral 2) Consultation 3) Ovarian stimulation Protocols 4) Stimulation Cycle Monitoring 5) Oocyte Retrieval 6) Postoperative Care 7) Collaborative Care. This framework is a starting point that should be adapted and improved upon by a larger, more compressive collaboration of subject matter experts for standard of care guidelines. Our goal in bundling lessons from our institution’s experience with fertility preservation and oocyte cryopreservation is to help expand the comfort of providers and thereby access to care for AYA transmen who desire fertility preservation until standard guidelines are expanded. There are many ways to build families for gender nonconforming individuals, and fertility preservation may not be the right option for all AYA transmen. However, it is crucial to enable gender affirming youth the opportunity to be educated about their fertility options for utilizing their own gametes.

The primary strength of our study is its inclusion criteria of a greater than 10 year time span, allowing for the largest published case series on transgender adolescents undergoing oocyte cryopreservation. This study broadly assesses multiple oocyte cryopreservation cycles to gain insights into standardized practices tailored towards AYA transmen. Our study is limited in its generalizability as it was completed at a single institution. Furthermore, our population was limited in their prior exposure to pubertal blockers and testosterone, making it difficult to draw conclusions on this specific patient population. More comprehensive and expansive research is needed to evaluate the outcomes and experiences of transmen who are on testosterone or pubertal blockers. Additionally, its retrospective design and reliance on chart documentation limited our ability to further explore decision-making surrounding fertility preservation, barriers to consultation or fertility preservation, regret/emotional stress/physical comfort during consultation or oocyte cryopreservation, and satisfaction with the process. In particular, we were not set up to use standard questions and instead relied on chart review of primary care, social work, and infertility specialist documentation surrounding episodes of gender dysphoria, expressed concerns with fertility goals, and reasons for declining fertility consultation or preservation. This methodology is limited in our ability to capture gender dysphoria episodes or reflect the complicated decision-making surrounding the OC process. This study focused on patients who pursued formal REI consultation and may be skewed towards those more likely to undergo oocyte cryopreservation. Further oocyte cryopreservation outcomes related research is needed to provide evidence-based and patient-centered care surrounding AYA fertility preservation. Further work is needed to standardize and tailor oocyte cryopreservation protocols towards the unique needs of AYA transmen, including the development of pubertal pathways as well as pathways for those previously exposed to testosterone or pubertal blockers. Finally, and possibly most importantly, the young nature of oocyte cryopreservation in this population has limited outcome data from oocyte utilization and fertilization and so the true reproductive potential of these cryopreserved gametes is unknown.

In conclusion, we present the largest published case series of oocyte cryopreservation in AYA transmales and identify lessons learned for best practices based on the experiences of our AYA transmale patients, as a tool for AYA patients and healthcare professionals caring for transgender and gender-nonconforming adolescent and young adults. These lessons may help inform more standard guidelines to empower both patients and providers to better understand fertility consultation, fertility preservation, and oocyte cryopreservation, provide transparency surrounding the oocyte cryopreservation process, and ease the decision to pursue an oocyte cryopreservation cycle in parallel to their gender-affirmatory care.

## Data Availability Statement

The raw data supporting the conclusions of this article will be made available by the authors, without undue reservation.

## Ethics Statement

The studies involving human participants were reviewed and approved by New York University Grossman School of Medicine Institutional Review Board (i13-00389). Written informed consent from the participants’ legal guardian/next of kin was not required to participate in this study in accordance with the national legislation and the institutional requirements.

## Author Contributions

JB and MF conceived and designed the study. FB completed the statistical analysis and drafted the manuscript. JS gave critical revision for important intellectual content. All authors interpreted the data, revised the paper for important intellectual content, and approved the final version. All authors contributed to the article and approved the submitted version.

## Conflict of Interest

The authors declare that the research was conducted in the absence of any commercial or financial relationships that could be construed as a potential conflict of interest.

## Publisher’s Note

All claims expressed in this article are solely those of the authors and do not necessarily represent those of their affiliated organizations, or those of the publisher, the editors and the reviewers. Any product that may be evaluated in this article, or claim that may be made by its manufacturer, is not guaranteed or endorsed by the publisher.
