# Supplementary material for: Fertility Preservation for Adolescent and Young Adult Transmen: A Case Series and Insights on Oocyte Cryopreservation
Source: Front Endocrinol (Lausanne). 2022 May 24;13:873508. doi: 10.3389/fendo.2022.873508 (PMC9171925; doi:10.3389/fendo.2022.873508)
Supplement: Supplementary file 1 [file Table_1.docx]

Supplemental Chart A – Oocyte Cryopreservation Outcomes Based on Prior Exposure to Leuprolide, Testosterone, and Oral Contraception

|  |  |  | **P value** |
| --- | --- | --- | --- |
|  | No Prior Gender Affirming Medications  (n=15) | Any Gender Affirming Medication (Oral contraception, Testosterone, and/or Leuprolide)  (n=6) |  |
| Age at Stimulation (years) | 15 (12-19) | 16.5 (14-25) | **0.02** |
| Anti-mullerian Hormone (ng/ml) | 3 (0.6-53) | 2.7 (0.44-7.35) | 0.50 |
| Follicle stimulating hormone (mIU/mL), cycle day 2 | 5 (1.7-9.4) | 7.9 (2.3-9.5) | 0.40 |
| Estradiol (pg/mL), cycle day 2 | 43 (20-163) | 37.5 (<12-48) | 0.47 |
| Total Gonadotropin Dose (IU) | 2350 (825-5400) | 2287.5 (1900-4500) | 0.86 |
| Duration of Stimulation (days) | 10 (8-13) | 11 (9-11) | 0.49 |
| Maximum Estradiol Level (pg/mL) | 3151 (1059- 6091) | 4155.5 (2673-6969) | 0.33 |
| Oocytes retrieved, number | 22 (18-43) | 23.5 (9-59) | 0.73 |
| Metaphase II (MII) oocyte cryopreserved, number | 15 (3-26) | 23.5 (8-35) | 0.57 |
| Metaphase I (MI) oocyte cryopreserved, number | 2 (0-8) | 0.5 (0-9) | 0.21 |
| Germinal Vesicle (GV) oocyte cryopreserved, number | 3 (0-20) | 2.5 (0-20) | 0.68 |
